# Memristive circuit-based model of central pattern generator to reproduce spinal neuronal activity in walking pattern

**DOI:** 10.3389/fnins.2023.1124950

**Published:** 2023-02-28

**Authors:** Dinar N. Masaev, Alina A. Suleimanova, Nikita V. Prudnikov, Mariia V. Serenko, Andrey V. Emelyanov, Vyacheslav A. Demin, Igor A. Lavrov, Max O. Talanov, Victor V. Erokhin

**Affiliations:** ^1^ITIS, IFMB, Kazan Federal University, Kazan, Russia; ^2^B-Rain Labs LLC, Kazan, Russia; ^3^National Research Centre Kurchatov Institute, Moscow, Russia; ^4^Moscow Institute of Physics and Technology, National Research University, Moscow, Russia; ^5^Department of Neurology, Mayo Clinic, Rochester, MN, United States; ^6^Skolkovo Institute of Science and Technology, Moscow, Russia; ^7^Institute for Artificial Intelligence R&D, Novi Sad, Serbia; ^8^Consiglio Nazionale delle Ricerche at Istituto dei Materiali per l'Elettronica ed il Magnetismo, Rome, Italy

**Keywords:** central pattern generator (CPG), memristive neuron, organic memristive device, spinal cord, leaky integrate-and-fire (LIF) neuron, walking pattern formation

## Abstract

Existing methods of neurorehabilitation include invasive or non-invasive stimulators that are usually simple digital generators with manually set parameters like pulse width, period, burst duration, and frequency of stimulation series. An obvious lack of adaptation capability of stimulators, as well as poor biocompatibility and high power consumption of prosthetic devices, highlights the need for medical usage of neuromorphic systems including memristive devices. The latter are electrical devices providing a wide range of complex synaptic functionality within a single element. In this study, we propose the memristive schematic capable of self-learning according to bio-plausible spike-timing-dependant plasticity to organize the electrical activity of the walking pattern generated by the central pattern generator.

## 1. Introduction

We consider as fundamental the problem of the creation of a new type of hardware as bio-compatible self-organizing electronic schematic of the part of the nervous system. The self-organization of the nervous system is crucial for the process of learning and rehabilitation including neuro-rehabilitation. The scale of the problem is significant according to World Health Organization statistics: the yearly increment of patients with spinal cord injury (SCI) is from 250,000 to 500,000 worldwide. The frequency of SCI is 2–5 for 100,000 of the urban population in the U.S. The increase in the number of patients with SCI only in the US is 10,000 per year (National, 2014).

Current approaches include cutting-edge technologies for the invasive and non-invasive stimulation using implant programmed by neuro-rehabilitation specialists with no option for the device self-adaptation and compensation for the particular medical case properties (Mikhaylov et al., 2020). The creation of biocompatible electronic circuit, which is capable of self-learning *via* spike-timing-dependant plasticity (STDP), seems perspective for the SCI treatment. STDP is a biological process of synaptic connection adjustment, whose strength depends on timing between pre- and postsynaptic spikes. Previously, it has been proved the possibility of using organic memristive devices as basic elements of self-organized neuromorphic systems (Erokhin, 2020) and applying them to the memristive synaptic prosthesis (Juzekaeva et al., 2018). These works lay the foundation for use of the memristive devices in the field of medical neurostimulation and implementation of biocompatible and adaptive neuronal electronic circuits. In the current work, we demonstrate the power of self-organization *via* bio-plausible STDP in polyaniline (PANI) memristive devices (Supplementary Figure S3) for the organization of the resembling shape of delays of responses.

The possible application domains of the work can be connected to the neurorehabilitation of the SCI, in this medical case, with the potential to replace part of the neural circuitry with memristive circuitry. The applied research of the neuromorphic and neurostimulation technology in the medical domain is at an early stage of development (Mikhaylov et al., 2020), and the topology of the spinal central pattern generator (CPG) itself is still under research (Rybak et al., 2015). Recently, we published works introducing a computational bio-compatible approach incorporating Oscillator Motif (OM)—the basic neuronal generator (Leukhin et al., 2020; Talanov et al., 2020). Later, we extended the use of OMs for the neurointerfaces (Talanov et al., 2021) and spasticity compensating devices (Mikhailova et al., 2022).

For the further development of neurostimulation-based neurointerfaces, especially taking into account the option to develop the implantable model-based neuroprosthesis, it is crucial to use energy-efficient devices. Memristive technology looks promising from this perspective, as they demonstrate fast-switching performance (~100 ps) (Torrezan et al., 2011), low power consumption (1 fJ) (Li et al., 2022), excellent scalability (down to 2 nm feature size) (Pi et al., 2019), multilevel resistive switching (Matsukatova et al., 2022), and compatibility with the CMOS technology (Mikhaylov et al., 2020).

**Figure 1:**
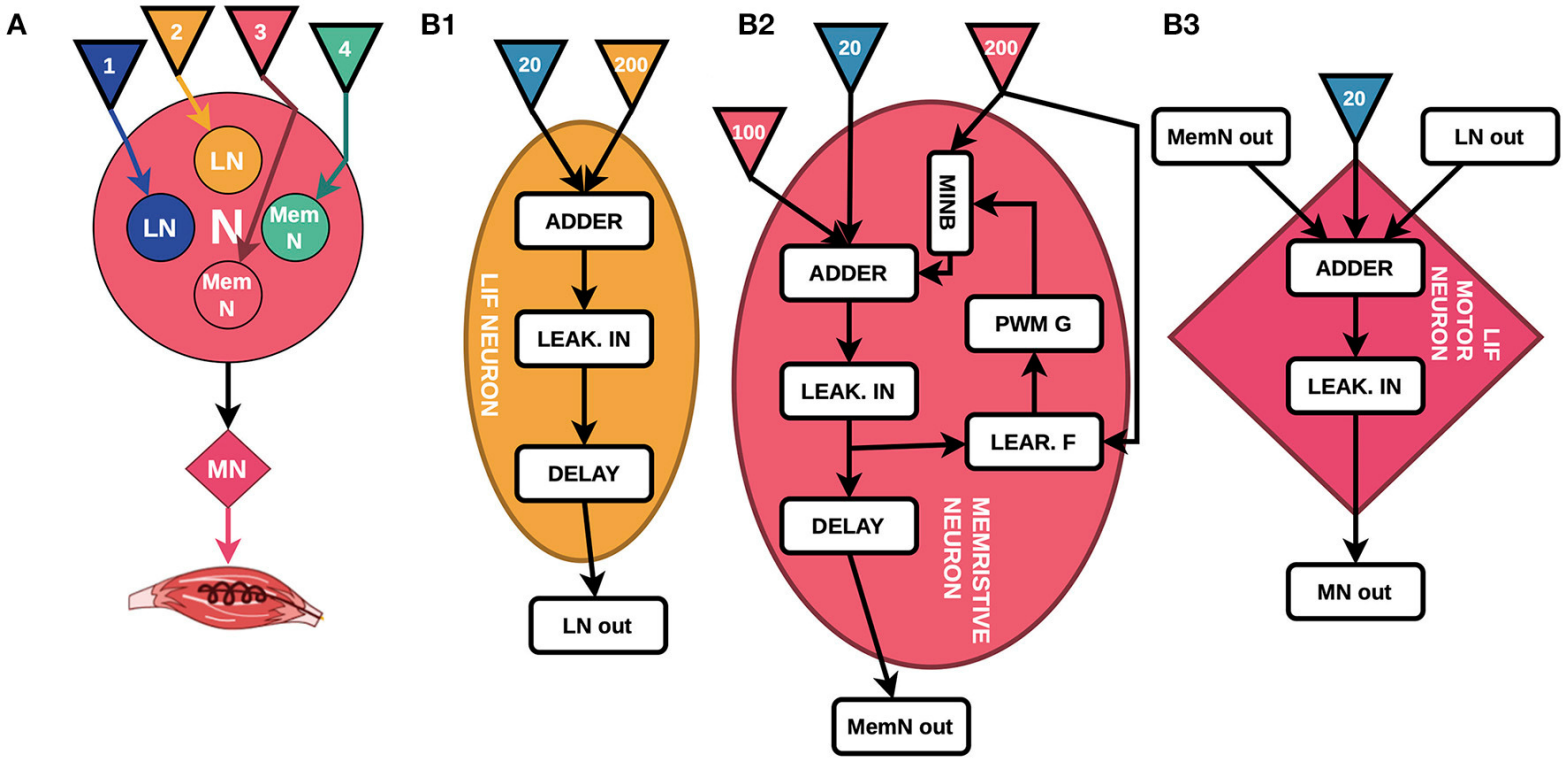
**(A)** High-level design diagram, where LNs are LIF neurons, MemNs are memristive LIF neurons, MN is a motor neuron, and colored triangles are sensory fibers coming from insole (numbers correspond to periods of 200 Hz pulses in Figures 2A2–A5). **(B)** The detailed block diagrams of all types of neurons. **(B1)** LIF neuron is the basis of MemNs and consists of Adder, Leakage Integrator (LEAK. IN), Delay, and inputs of 200 Hz (yellow triangle) and 20 Hz pulses (blue triangle). **(B2)** The principal schematic of MemN consisting of Learning Feedback (LEAR. F), Pulse-width modulation Generator (PWM G), Memristive device Integration Block (MINB), and additional 100 Hz pulses input (pink triangle). **(B3)** Motor neuron consisting of Adder that sums up signals from four neurons and 20 Hz pulses, LEAK. IN and Delay.

Further development of memristive bio-compatible devices controlling neuroprostheses is inspired by early works (Lavrov et al., 2006; Gad et al., 2013) on the neurorehabilitation of the complete SCI and simulation of the spinal circuitry (Rybak et al., 2015). In the current work, we propose a new type of self-organizing hardware implemented as a minimalist schematic consisting of five neurons with memristive circuits in two of them generating electrical responses resembling delays structure in the electrical output of modulated motor evoked response triggered by the epidural electrical stimulation (EES) between early responses (10 ms) and late responses (later than 30 ms) during locomotion observed in biological studies (Lavrov et al., 2008a,b; Gad et al., 2013). The bio-plausible setup of the delays structure is done using the self-learning process implemented in the memristive neurons *via* learning feedback (Figures 1, **3**).

## 2. CPG circuit

**Figure 2:**
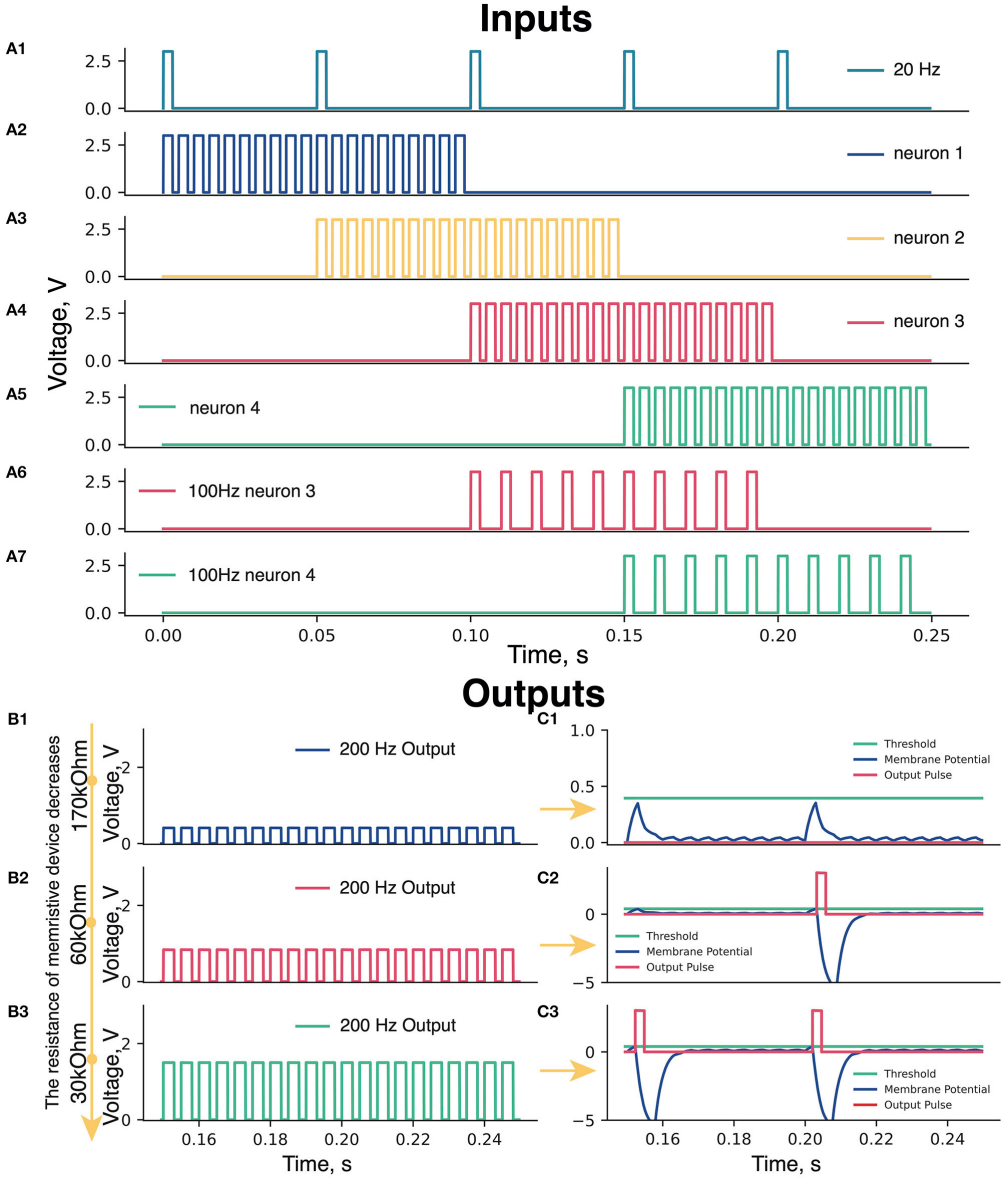
**(A)** Input pulses. **(A1)** 20 Hz pulses; **(A2–A5)** Periods of 200 Hz input pulses: blue (0–100 ms), yellow (50–150 ms), pink (100–200 ms), and green (150–250 ms) triangles (Figure 1). **(A6, A7)** Periods of 100 Hz for pink (100–200 ms) and green neuron (150–250 ms). **(B)** Amplitudes of 200 Hz signals at different resistances of the memristive device in green neuron. Memristive device resistance **(B1)** 170 kOhm, **(B2)** 60 kOhm, and **(B3)** 30 kOhm. **(C)** The membrane potential of the Leakage Integrator with memristive device various resistances. **(C1)** 170 kOhm—low amplitude of 200 Hz pulses—the total sum of the 200 and 20 Hz signals cannot raise the voltage on the Leakage Integrator (blue curve) to the threshold value (green line)—no output signals from the Leakage Integrator (red line). **(C2)** 60 kOhm—sum of pulses of 200 and 20 Hz is enough to take the voltage to the threshold value once and trigger an output pulse (red curve)—refractory period of the Leakage Integrator [voltage below 0 V (200–215 ms)]. **(C3)** 30 kOhm—the sum of two signals of 200 and 20 Hz is enough to cause the output pulses of the Leakage Integrator two times.

We present the overall high-level view of the system in Figure 1A, where N stands for the nucleus that forms the delays structure and MN stands for motor neuron. The inputs of the memristive devices-based four neurons system are sensory inputs from the insole represented as arrows blue, yellow, pink, and green, colors correspond to those of the input pulse trains in Figure 2A; motor neurons of extensor (MN, implemented in the work), presented in details in Figure 1B, generates output that could be integrated into the muscle of the biological model. Figure 1B shows a general block diagram describing the principles of operation of the CPG schematic. The presented diagram is the two nuclei structure containing four leaky integrate-and-fire (LIF) neurons (Abbott, 1999) where two of them use memristive devices (MemNs in Figure 1A), while other two have only resistors (LNs in Figure 1A) as input weights in pattern formation nucleus N and one LIF motor neuron MN.

### 2.1. Input signals of 200 and 20 Hz

The input signal of 200 Hz (Handler and Ginty, 2021) emulates sensory feedback from a foot. We assume that sensory inputs from heel to toe triggers neurons in pattern formation nuclei in the manner presented in Figures 2A2–A5. The role of the modulation of motor-evoked responses during stepping facilitated with EES was suggested in earlier animal studies (Lavrov et al., 2006, 2008b; Gad et al., 2013). Recent studies indicated the use of the EES within a range of 15–30 Hz stimulation frequency for the recovery of motor functions in humans (Gill et al., 2018; Wagner et al., 2018). The input signal of 20 Hz (Figure 2A1) was applied to the neurons to simulate the EES.

### 2.2. What is a 100 Hz signal used for?

Before starting the learning process, the memristive device is in non-conducting state. Therefore, the voltage at the Leakage Integrator (scheme in Supplementary Figure S4) does not reach the threshold value and no output pulses are generated. In this situation, memristive device learning is impossible, since the output pulses are assumed to trigger the Learning Feedback (scheme in Supplementary Figure S5). To solve the problem of 0-output, the 100 Hz input is applied to the circuit (Figures 2A6, A7). It raises the membrane potential in the Leakage Integrator to exceed a threshold value and causes output pulses generation, which in turn triggers the initial learning of the memristive device.

This 100 Hz signal can be considered as an additional drive for stimulation of exclusively artificial neurons. It is likely that such an additional type of internal stimulation would not be needed when one would scale the proposed scheme up to several hundreds or thousands of memristive neurons, due to fine-tuning of their parameters (e.g., lowering the threshold voltage). This is a matter for future research.

### 2.3. PANI-based memristive device

For our purpose, we used a standard PANI-based memristive device operating in the two-terminal mode (with drain and gate terminals connected as an output). Even if the device has three terminals, it cannot be considered as “transistor” because the potential on the third electrode is fixed and the resistance switching is due to the ionic charge passed between it and conducting channel. The mechanism of resistance switching is connected to the redox reactions in the PANI channel, oxidized form PANI is highly conductive, while the reduced one is insulating (Berzina et al., 2009). The device parameters (device scheme, switching kinetics, endurance, operation in STDP, and high-frequency regimes) could be found elsewhere (Lapkin et al., 2018; Prudnikov et al., 2020; Gerasimov et al., 2021). The devices were fabricated according to the previously reported procedure (Prudnikov et al., 2020). Polyaniline (Mw = 10^5^ Da) 10-layer thin film were produced by Langmuir–Schaefer technique on a substrate with two gold electrodes with a gap of 10 μm. Then the polyethylene oxide (Mw = 600 kDa) electrolyte water solution was drop-casted onto the film. Silver wire (with a diameter of 50 μm) was placed in the solution and the device was exposed to drying in an airflow for 2 h. We have also proved the device is capable of changing resistance under signals of frequencies used in this work (Gerasimov et al., 2021). Memristive device sharply decreases the resistance when voltage above a certain threshold is applied and increases when the external voltage falls behind another threshold. In contrast, voltage amplitudes in the range between these threshold values do not show a significant effect and could be used for reading the resistive state. Current–voltage curves of the devices operating in the three-terminal mode are shown in Supplementary Figure S3. It is to underline that the used device has a very important advantage with respect to widely used filament formation-based memristive systems: values of switching voltages are fixed which is very important for neuromorphic applications.

### 2.4. Memristive neurons

All pulses of 200 Hz signals pass through the memristive device integration block (scheme in Supplementary Figure S1), where the amplitudes of these signals change depending on the resistance of the memristive device in each neuron. In the case of high memristive device resistance, the output of the memristive device integration block initiated by 200 Hz pulses has a small amplitude, whereas with a low resistance, the amplitude is higher. An example of changing the amplitude of 200 Hz pulses depending on the different resistances of the memristive device is shown in Figure 2B. Figure 2B1: R_*mem*_ = 170 kOhm – A_200*Hz*_ = 0.4*V*; Figure 2B2: R_*mem*_ = 60 kOhm—A_200*Hz*_ = 0.85*V*; Figure 2B3: R_*mem*_ = 30 kOhm—A_200*Hz*_ = 1.6*V*).

### 2.5. Adder and leakage integrator blocks

Each pulse at a neuron (blue and yellow: 200 Hz and 20 Hz; pink and green: 200 Hz, 20 Hz, and 100 Hz) enters the Adder block (scheme in Supplementary Figure S2), where they are summed up and the resulting signal is further transmitted to the input of the Leakage Integrator. The Leakage Integrator performs three functions: (1) update of the membrane potential of the neuron when exposed to input pulses; (2) generation of the output pulse when the membrane potential in the Leakage Integrator reaches the threshold value; (3) reduction of the membrane potential of the neuron to negative values after generating output pulses (refractory period). Examples of the operation of the Leakage Integrator when the membrane potential does not and does reach the threshold value are shown in Figures 2C1, C2, respectively.

### 2.6. The operation of the leakage integrator at various resistances of the memristive device

As long as the resistance of the memristive device is high (170 kOhm), the 200 Hz signals are characterized by low amplitude, thus, the sum of the 200 Hz and 20 Hz signals in the Leakage Integrator cannot raise the neuron membrane potential to the threshold value, resulting in no output signals from the Leakage Integrator (Figure 2C1). With this resistance of the memristive device of 60 kOhm, the signal amplitude of 200 Hz input is sufficient for the membrane potential to reach the threshold value once and causes an output pulse (Figure 2C2). After the generation of the output pulse, the Leakage Integrator goes into a refractory period, during which the membrane potential value is below 0 V, and no pulse can raise this potential above zero. When the resistance of the memristive device approaches 30 kOhm, the sum of two signals of 200 and 20 Hz is sufficient to trigger the output pulses of the Leakage Integrator by every 20Hz pulse (Figure 2C3).

### 2.7. Learning feedback and PWM generator blocks

These blocks of the circuit are necessary for learning (tuning the memristive device resistance during the operation of the CPG circuit). As it was shown by the simulation (Suleimanova et al., 2021), two signals are required for the learning circuit to work—an input signal of 200 Hz and an output signal generated by the Leakage Integrator. Furthermore, based on the time difference (Δ*t*) between the rising edges of these pulses, the learning feedback circuit generates pulses according to the Hebbian learning function (see Supplementary Figure S5). Learning pulses are then transmitted to the pulse-width modulation (PWM) generator block (scheme in Supplementary Figure S6). Then, in this block, a PWM signal of a certain duty cycle, depending on the learning pulses (output from the Learning Feedback block), is generated. The resulting PWM signals change memristive device resistance in the direction of either decrease or increase based on a learning rule.

### 2.8. Delay and motor neuron

Output signals generated by the Leakage Integrators are then transmitted to the Delay block (scheme in Supplementary Figure S7), which makes a delay in further signal transmission for a certain amount of time. The role of the Delay block is to simulate synaptic delays between LNs, MemNs, and the motor neuron. The output signal of the Leakage Integrator at the blue and green neurons have the lowest and highest delays, respectively. The output pulses from the Delay blocks are then sent through the Adder, where they are summed up, to the Motor Neuron (scheme in Supplementary Figures S8, S9). In the latter, block final membrane potential is formed. The wiring diagram of the implemented CPG circuit is presented in Supplementary Figure S10.

## 3. Results

**Figure 3:**
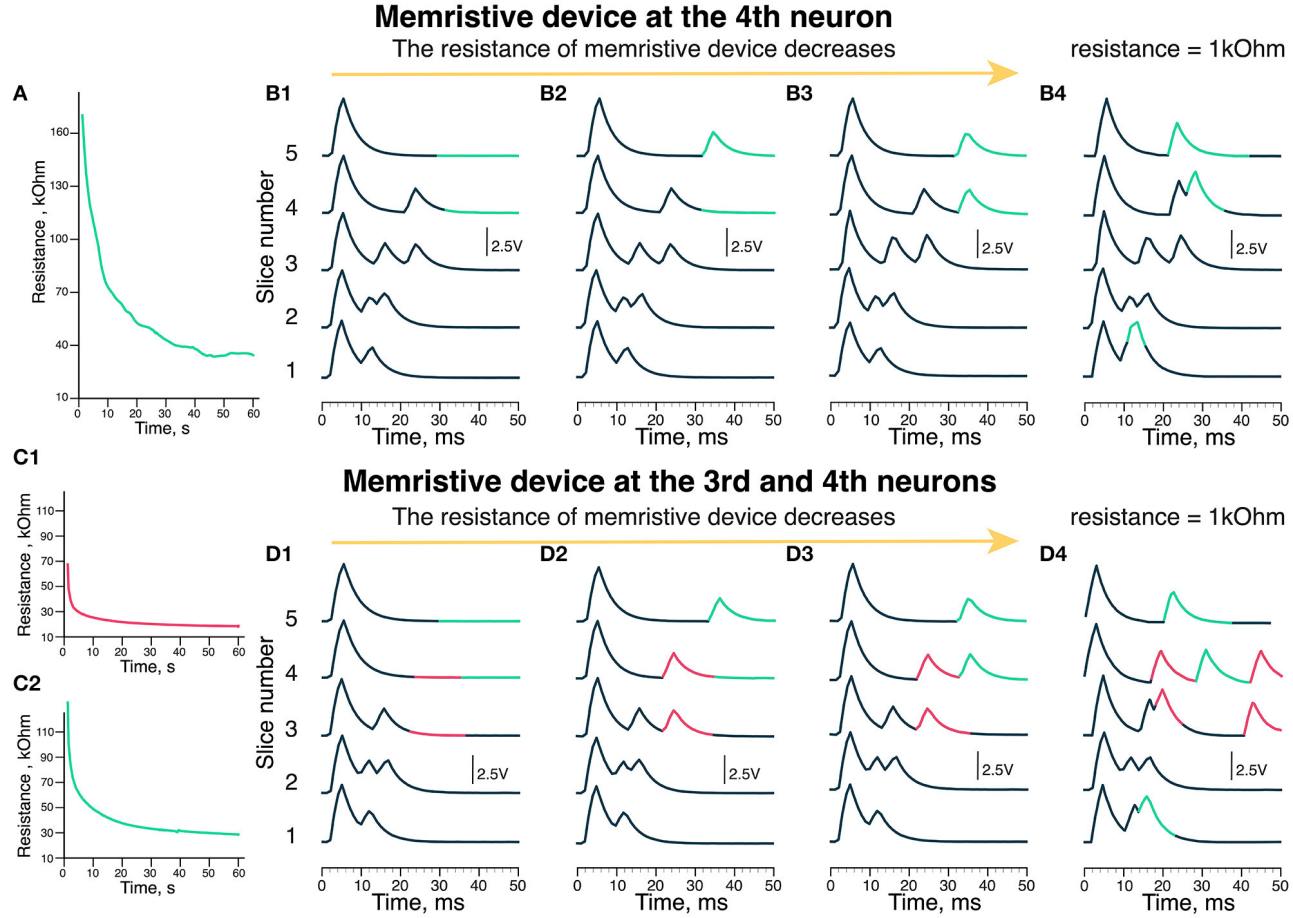
Output of the scheme (MN membrane potential). **(A)** The update of memristive device resistance in green neuron. **(B)** The recordings of output voltage with different memristive device resistance: **(B1)** 170 kOhm, **(B2)** 60 kOhm, **(B3)** 30 kOhm, and **(B4)** 1 kOhm (Resistor). **(C)** The update of memristive deice resistance in **(C1)** pink and **(C2)** green neuron of the scheme. **(D)** The recordings of output voltage with different memristive devices resistance: **(D1)** 70 kOhm in pink neuron and 120 kOhm green neuron, **(D2)** 30 kOhm in pink and 40 kOhm in green neurons, **(D3)** 20 kOhm in pink and 30 kOhm in green neurons. **(D4)** 1 kOhm (Resistor) in pink and 1 kOhm (Resistor) in green neurons. **(B4, D4)** Are the experiments where we used resistors instead of memristive devices to reproduce the situation of extremely low resistance.

We have implemented and tested two nuclei memristive circuits (Figure 1A) in two modes: with one reconfigurable neuron green (pink neuron input resistance was set to be optimal for the delays structure formation) and with two reconfigurable neurons (pink and green) while the other neurons had fixed weight connections. Figure 3A indicates the changes in the memristive device resistance of green neurons during the learning process in the first mode. Figures 3B, D shows recordings of the MN potentials with different resistance values of the memristive device in green neuron. The recordings in Figures 3B4, D4 show neuron potential with 1 kOhm resistance in the input channel.

During the experiments, the resistance of the memristive device in green neuron decreased from 170 to 30 kOhm (Figure 3A). In the first case (Figure 3B1), the resistance of the memristive device is too high (170 kOhm), thus there are no responses later than 30 ms (highlighted in pink) since the amplitude of 200 Hz input is not sufficient to produce an output signal after passing the memristive device. Thus, the green neuron's potential does not reach the threshold of the Leakage Integrator excited by 20 and 200 Hz signals.

In the next case shown in Figure 3B2, a later than 30 ms response is triggered by green neuron is formed during the last 30 ms of the experiment (pink). The resistance of the memristive device is 60 kOhm, while there is no late (30 ms) response in the fourth slice. Thus, the potential of the green neuron reaches the threshold but it takes more than 50 ms and two series of 20 and 200 Hz inputs with 60 kOhm resistance. When the resistance of the memristive device becomes <40 kOhm (30 kOhm in Figure 3B3 case) that corresponds to modulation of delays in motor evoked potentials formed during EES facilitated locomotion (Gad et al., 2013; Figure 3B3). The late responses are produced in the 30–40 ms time frame (highlighted in pink).

Figure 3D shows the output of the circuit with two memristive neurons. Figure 3C1 shows the change in the memristive device resistance at the pink neuron of the circuit, where the initial resistance is 70 kOhm. The resistance of this memristive device decreased to 20 kOhm during the experiment. Figure 3C2 shows the update of the memristive device resistance as the input of green neuron, where the initial resistance of the memristive device is 120 kOhm. By the end of the experiment, the resistance of this memristive device decreased to 20 kOhm. Initially, there are no responses on the third to fifth slices with these memristive devices high resistance (Figure 3D1). With resistance values in the range of 70–120 kOhm, the membrane potential does not reach the threshold to generate an output signal.

The second recording in Figure 3D2 shows neuron potential where responses later than 30 ms are evoked by the outputs of pink neuron (highlighted in pink) and green neuron (highlighted in green) at 3–5 slices. The initial resistance was different for the memristive devices of pink and green neurons, so the memristive device of pink neuron learned (decreased resistance) faster. Thus, in Figure 3D2, there are two late responses evoked by pink neuron (pink) and one late response evoked by green neuron (green). The resistance of the memristive device in the leakage input in the pink neuron was 30 kOhm, while in the green one, it was 40 kOhm.

Figure 3D3 shows the third recording of the potential, where both pink and green neurons generate two output signals. The memristive device resistance in the leakage input in pink neuron was 20 kOhm and 30 kOhm in green neuron. In the 2nd recording (Figure 3D2), resistance of the neuron input at the pink neuron equals to 30 kOhm and two output signals are generated. Thus, with the optimal resistance of the memristive device in the range of 20–30 kOhm like in Figure 3D3. The optimization of the learning parameters is done by the threshold of memristive neuron.

We conducted a series of experiments with 1 kOhm resistor since the memristive device was not able to reach that resistance in this exact experiment (however, this value is within the operating range of the PANI-based memristive device). In Figure 3B4, with a low resistance of 1 kOhm at the input of green neuron, responses are produced earlier at slices 4 and 5 than in Figure 3B3 with a memristive device. In addition, there is a response right after the early (10 ms) response at the 1st slice. In Figure 3D4, with low resistance (1 kOhm), at the input of pink and green neurons, the responses appear chaotically. There are two responses produced by pink neuron at slices 3 and 4. The responses produced by green neuron are similar to Figure 3B4. This resistance is too low and one stimulus from 200 Hz input is enough to reach a threshold and generate an output signal of the leakage in neurons. It means that using memristive devices is preferable for such a task.

## 4. Discussion and conclusion

We proposed a new type of hardware implementation of a primitive electronic circuit with memristive devices producing the resembling biological delays structure in electrical output modulation of motor evoked response during locomotion facilitated with EES. The delays are set by the resistance of memristive devices replicating the learning dynamics of biological synapses representing the overall conductance of neuron synapses, including the creation and destruction of synapses and changes in their individual conductance. The resistance of the memristive device changed due to the proper conformity of input EES pulses and feedback ones according to the STDP rule. During the experiments, we defined the following ranges of memristive devices resistance value: (1) higher than 70 kOhm—no output signal of neuron, resistance is too high to produce an output signal; (2) 60–40 kOhm—the schematic generates one output signal of neuron per 100 ms, i.e., once for five input stimuli; (3) 30–20 kOhm—the optimal resistance to produce the output signals with the desired frequency, a potential of the LIF neuron reaches the threshold every time when input signals 20 and 200 Hz are intersected and summed up; (4) <1 kOhm—the pattern is ruined. The proposed schematic is capable to reproduce essential features necessary as the step forward to CPG reproduction in hardware. In future, we plan to integrate the produced schematic in the biological CPG for testing purposes.

## Data availability statement

The original contributions presented in the study are included in the article/Supplementary material, further inquiries can be directed to the corresponding authors.

## Author contributions

IL rework of biological justification and clearness of the article. DM and AS memristive neuron assembly and experimental work. DM, AS, MT, and VE results processing and article initial work. NP, MS, and AE PANI memristive devices fabrication and experiments with memristive neurons editing the article. VD overall scientific management of the project from the NRC Kurchatov Institute side. VE the overall management of the project. All authors contributed to the article and approved the submitted version.
