# Supplementary material for: Memristive circuit-based model of central pattern generator to reproduce spinal neuronal activity in walking pattern
Source: Front Neurosci. 2023 Feb 28;17:1124950. doi: 10.3389/fnins.2023.1124950 (PMC10011148; doi:10.3389/fnins.2023.1124950)
Supplement: Supplementary file 1 [file Presentation_1.pdf]

Memristive circuit-based model of central pattern generator to reproduce spinal neuronal activity in walking pattern

Figures S1,S2,S4-S8 show the electrical circuits of the elements in the block diagram, which is given in the article (**Figure 1 in the article).**

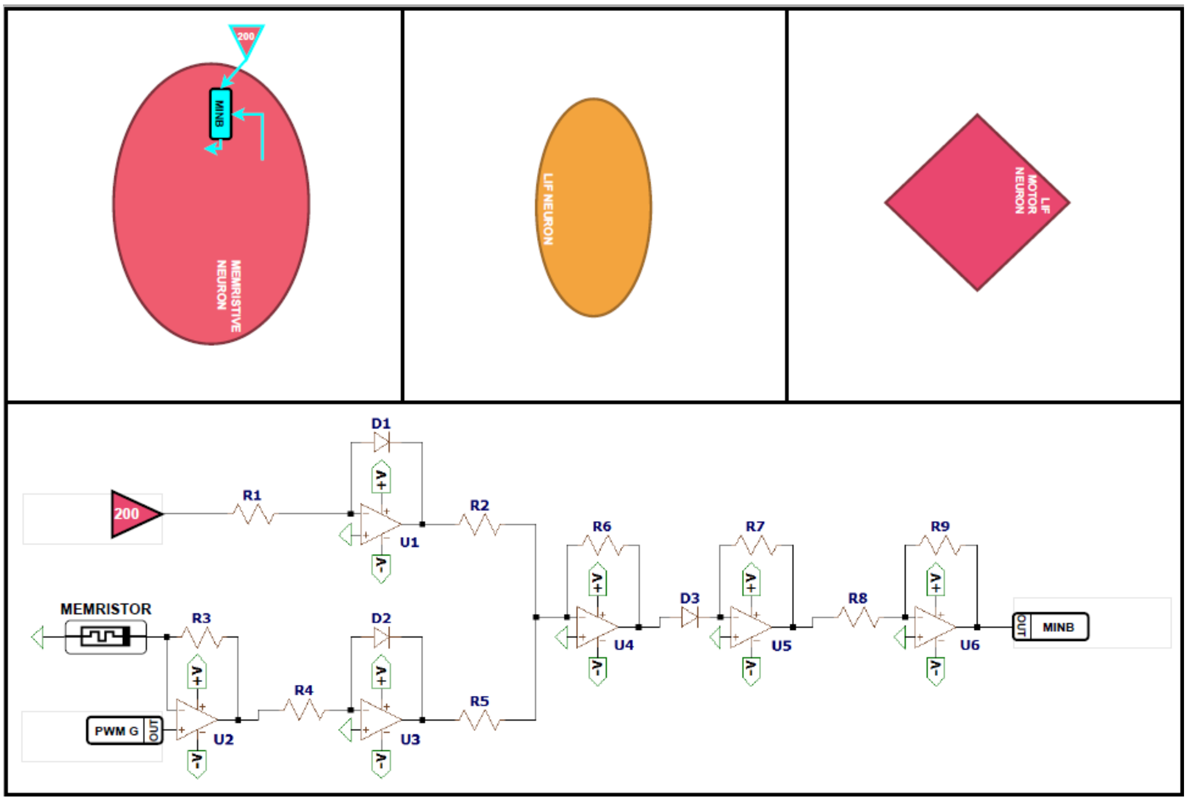

Figure S1. Memristive device Integration Block (MINB) schematic

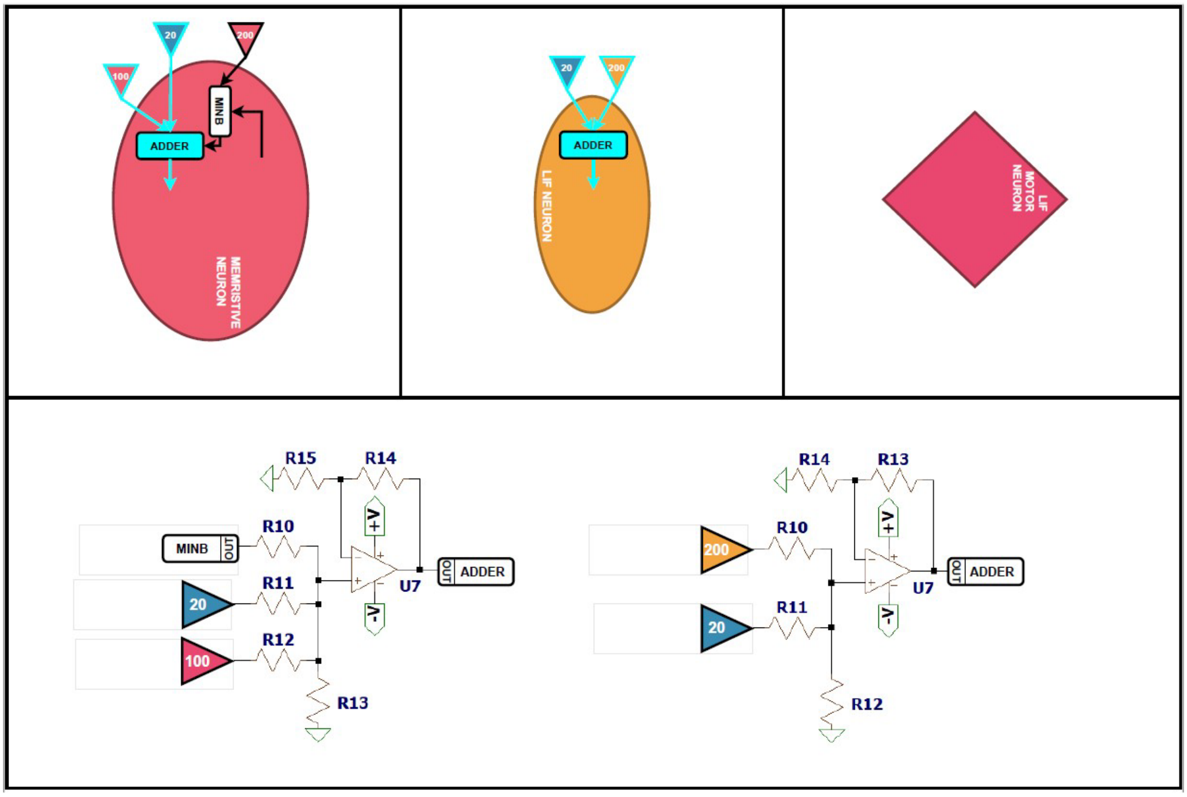

Figure S2. Adder schematic (ADDER)

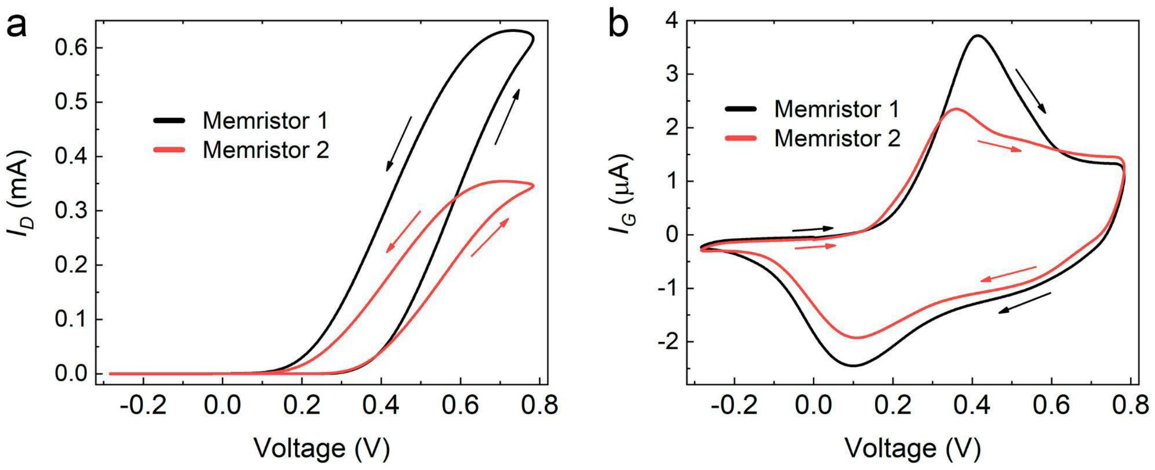

Figure S3. Current-voltage relationship of the two PANI memristive devices for drain (a) and gate (b) terminals. Memristive devices were fabricated using the same technique, but result in different IV-curves due to device-to-device variability. These two devices were used in CPG circuit realization.

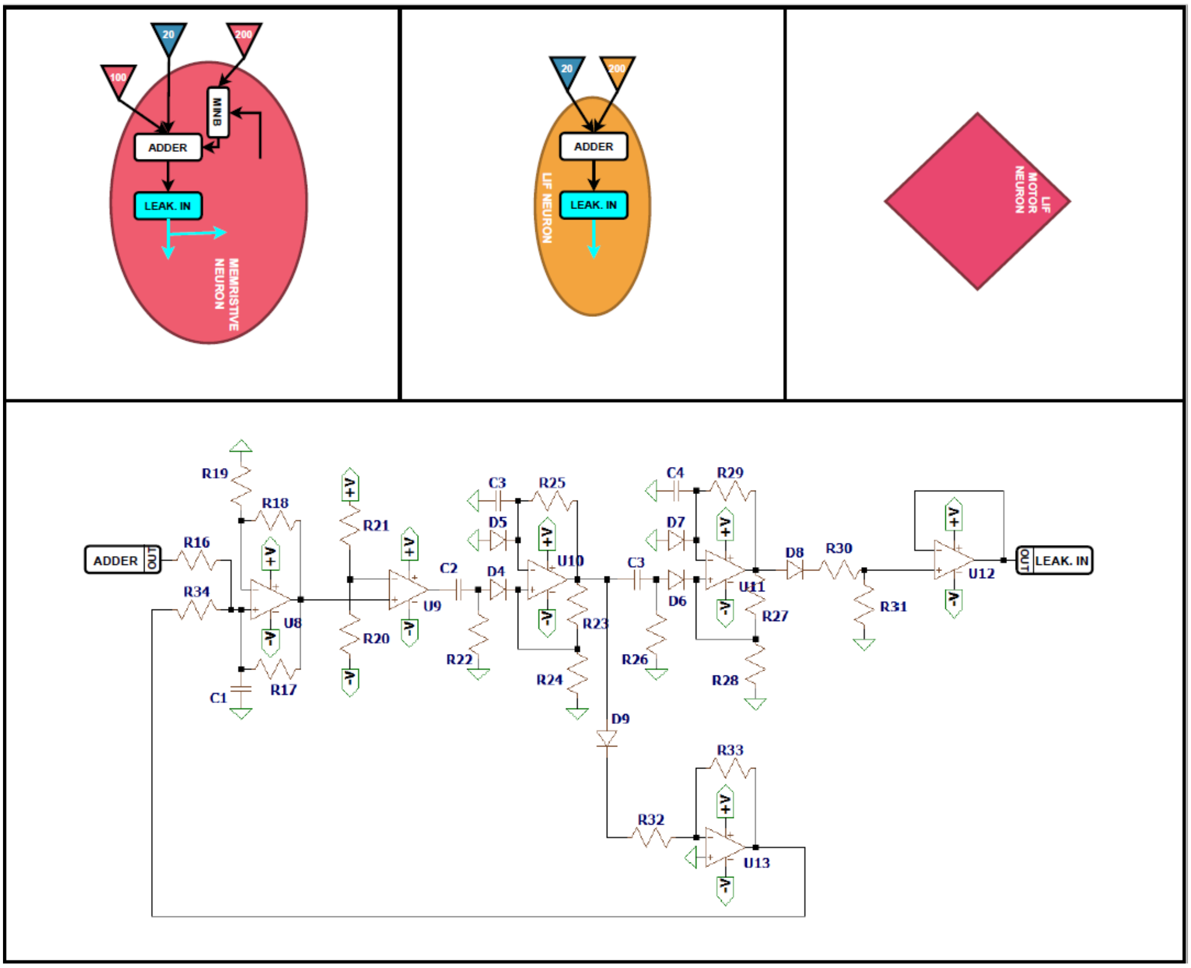

Figure S4. Leakage Integrator schematic (LEAK. IN)

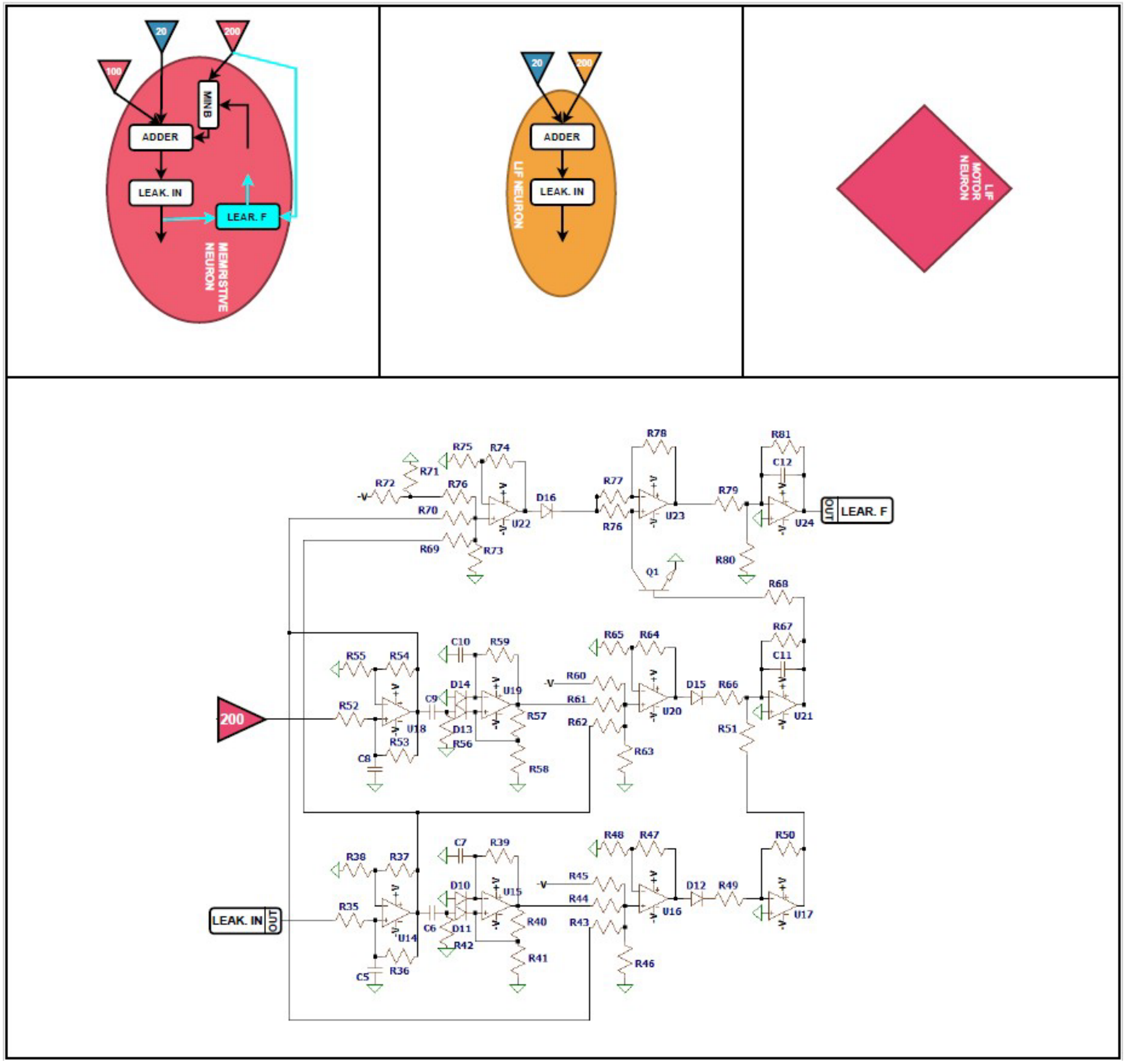

Figure S5. Learning Feedback schematic (LEAR. F)

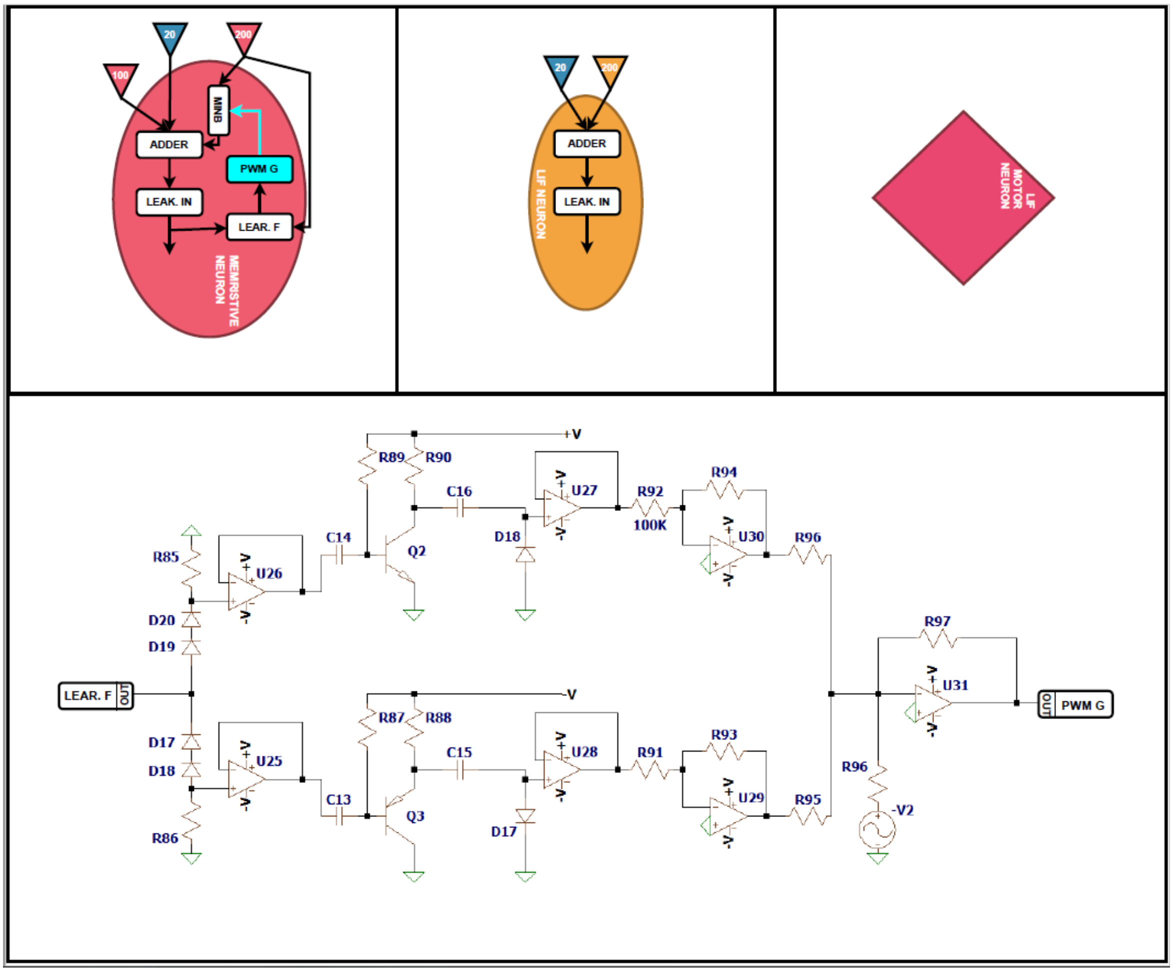

Figure S6. PWM Generator schematic (PWM G)

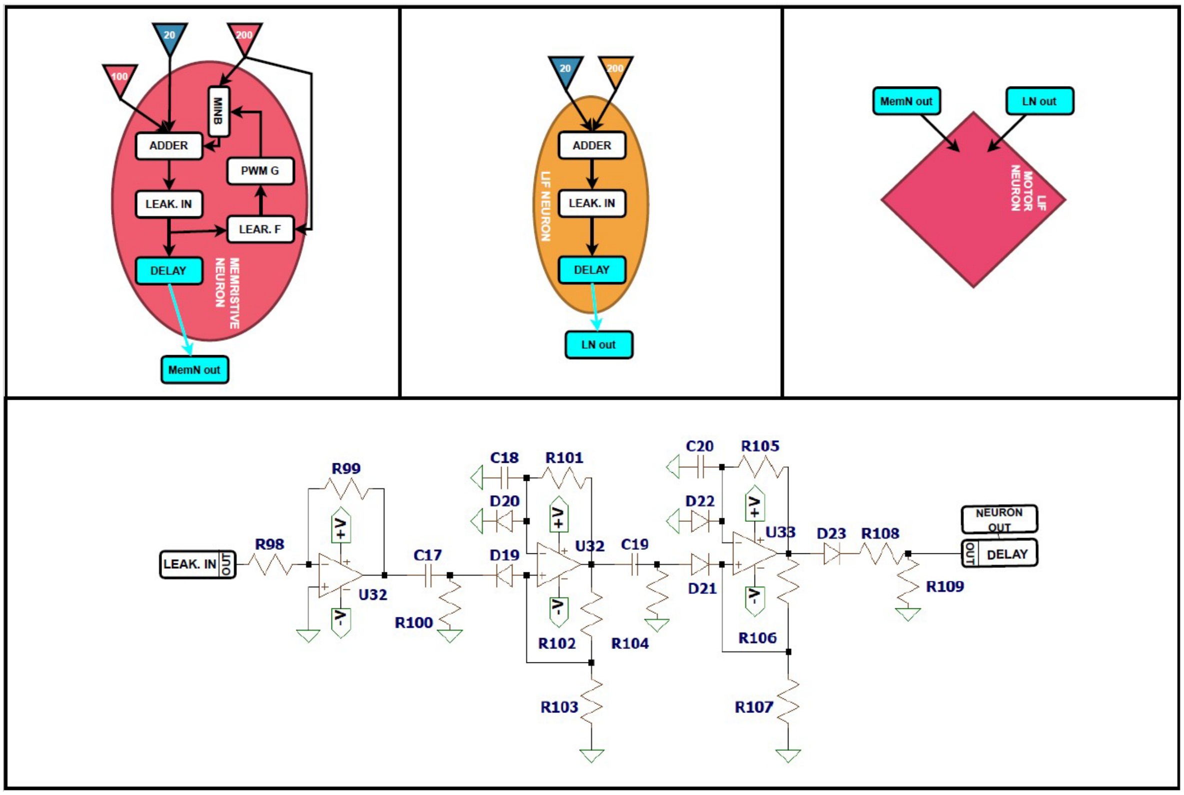

Figure S7. Delay of the memristive neuron output schematic (DELAY)

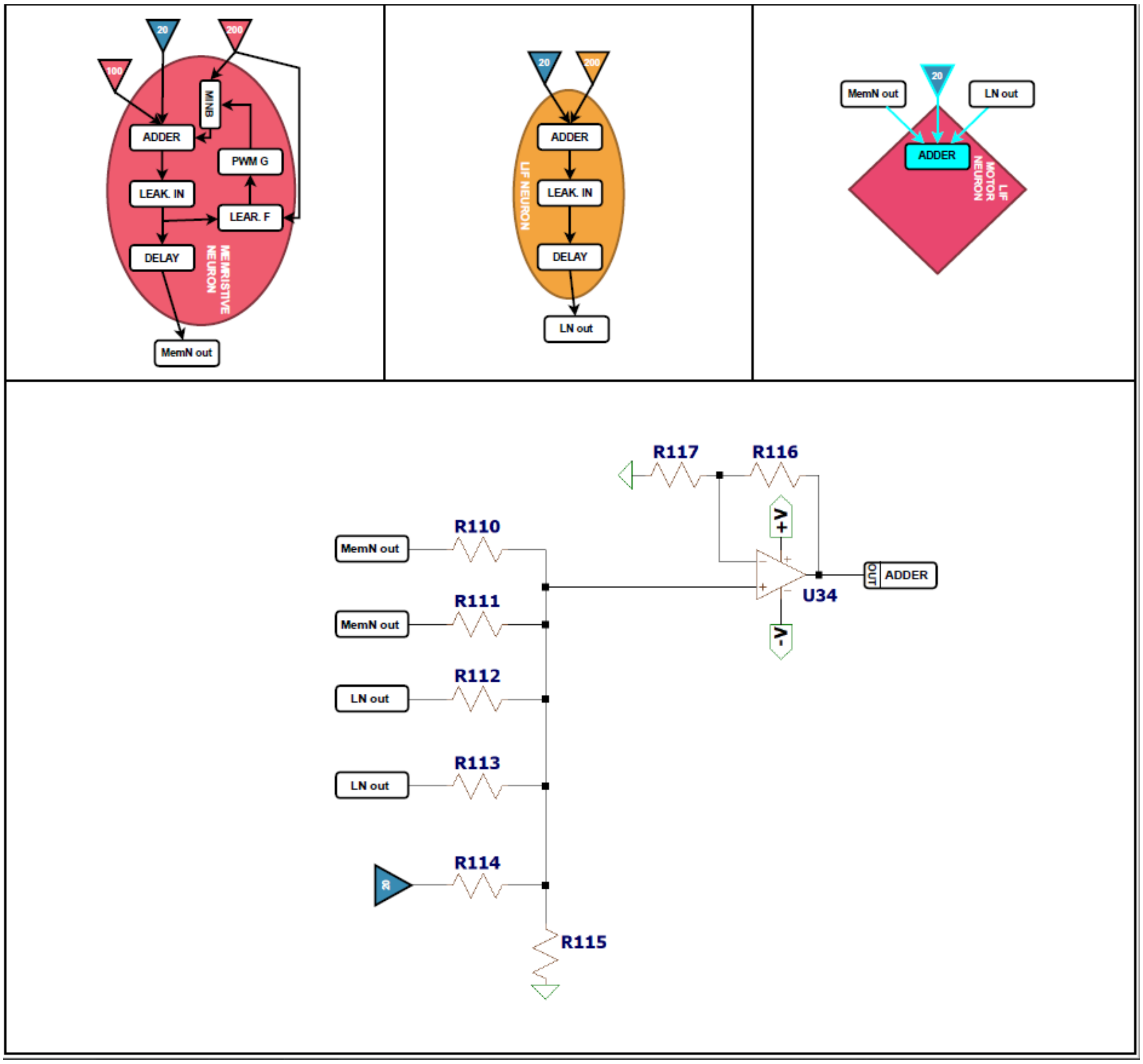

Figure S8. Adder of the motor neuron (ADDER)

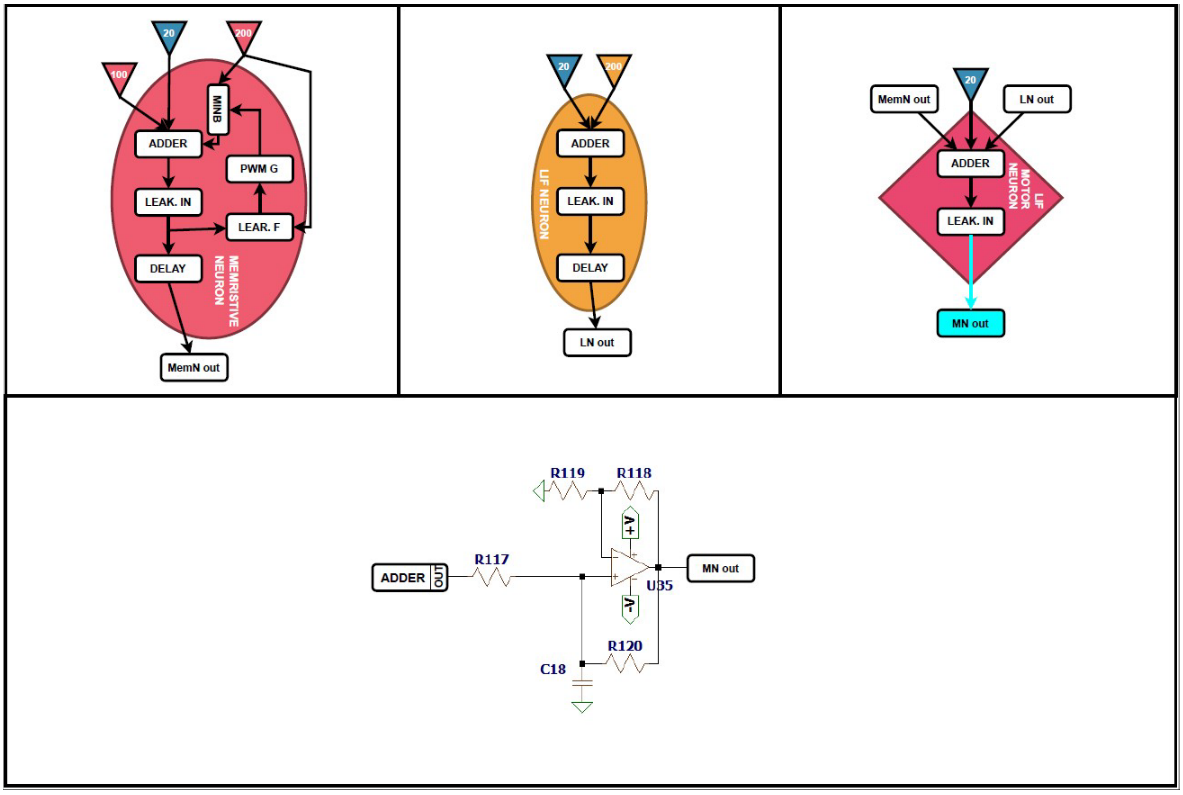

Figure S9. Leakage Integrator of the motor neuron (LEAK. IN)

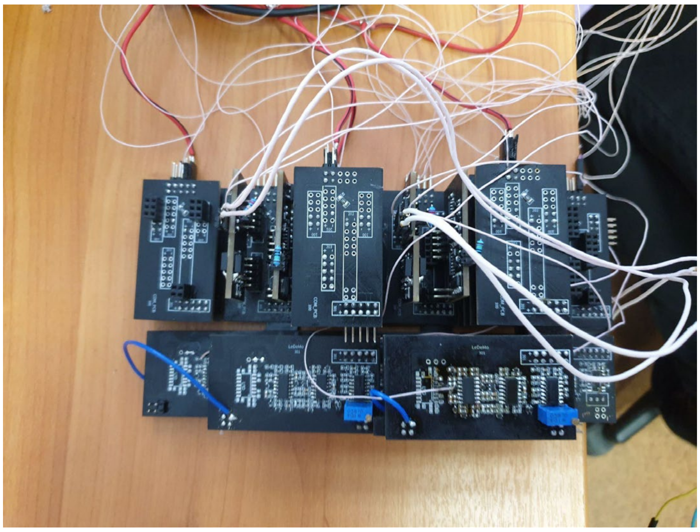

Figure S10. Top view of the implemented CPG circuit
